# Microglia and Aging: The Role of the TREM2–DAP12 and CX3CL1-CX3CR1 Axes

**DOI:** 10.3390/ijms19010318

**Published:** 2018-01-22

**Authors:** Carmen Mecca, Ileana Giambanco, Rosario Donato, Cataldo Arcuri

**Affiliations:** 1 Department of Experimental Medicine, Perugia Medical School, University of Perugia, Piazza Lucio Severi 1, 06132 Perugia, Italy; carmen.mecca@unipg.it (C.M.); ileana.giambanco@unipg.it (I.G.); rosario.donato@unipg.it (R.D.); 2 Centro Universitario per la Ricerca sulla Genomica Funzionale, Perugia Medical School, University of Perugia, Piazza Lucio Severi 1, 06132 Perugia, Italy

**Keywords:** aging, aged microglia, TREM2, DAP12, CX3CL1, CX3CR1

## Abstract

Depending on the species, microglial cells represent 5–20% of glial cells in the adult brain. As the innate immune effector of the brain, microglia are involved in several functions: regulation of inflammation, synaptic connectivity, programmed cell death, wiring and circuitry formation, phagocytosis of cell debris, and synaptic pruning and sculpting of postnatal neural circuits. Moreover, microglia contribute to some neurodevelopmental disorders such as Nasu-Hakola disease (NHD), and to aged-associated neurodegenerative diseases, such as Alzheimer’s disease (AD), Parkinson’s disease (PD), and others. There is evidence that human and rodent microglia may become senescent. This event determines alterations in the microglia activation status, associated with a chronic inflammation phenotype and with the loss of neuroprotective functions that lead to a greater susceptibility to the neurodegenerative diseases of aging. In the central nervous system (CNS), Triggering Receptor Expressed on Myeloid Cells 2-DNAX activation protein 12 (TREM2-DAP12) is a signaling complex expressed exclusively in microglia. As a microglial surface receptor, TREM2 interacts with DAP12 to initiate signal transduction pathways that promote microglial cell activation, phagocytosis, and microglial cell survival. Defective TREM2-DAP12 functions play a central role in the pathogenesis of several diseases. The CX3CL1 (fractalkine)-CX3CR1 signaling represents the most important communication channel between neurons and microglia. The expression of CX3CL1 in neurons and of its receptor CX3CR1 in microglia determines a specific interaction, playing fundamental roles in the regulation of the maturation and function of these cells. Here, we review the role of the TREM2-DAP12 and CX3CL1-CX3CR1 axes in aged microglia and the involvement of these pathways in physiological CNS aging and in age-associated neurodegenerative diseases.

## 1. Introduction

The existence of microglial cells within the CNS was demonstrated a century ago by del Rio-Hortega [1]. Despite the great amount of information about microglia, many characteristics of these cells have been controversial for a long time, including the identity of their precursors. To date, it is widely accepted that microglial cell precursors develop in the yolk sac [2], and Ginhoux et al. [3] additionally demonstrated that postnatal hematopoietic progenitors are not involved in the maintenance of adult microglia. However, the identity of microglial precursors and their gene expression profile are still unresolved issues. Similarly, it is still not completely understood if, during their lifespan, microglia renew in situ or if precursors originating outside the CNS participate in the renewal of the microglia population. In this context, studies proved that microglia maintenance is independent of bone marrow-derived progenitors during the adult life, and that CNS damages induce resident microglia proliferation [4].

Consistently, Solomon et al. demonstrated that, in a model of amyotrophic lateral sclerosis (ALS), microgliosis that occurs in the spinal cord is triggered by resident microglia and not by circulating precursors [5]. Recently, Réu et al. showed that, differently from other cells belonging to the hematopoietic lineage, microglia exhibited a median renewal rate of 28% per year, and many of these cells endured more than twenty years. Furthermore, the same authors did not identify quiescent long-lived microglia, concluding that these cells undergo a continuous and slow turnover throughout the adult life [6].

The terms M1 and M2 are widely used to define classically (proinflammatory) and alternatively activated (antiinflammatory) microglia, respectively, but the suitability of this terminology, originally referred to tissue macrophages, is debated [7]. The term M1 and M2 will be used here to denote proinflammatory and antiinflammatory phenotypes, respectively, for the sake of simplification. In physiological conditions, microglia exhibit an expression profile characterized by specific morphologic features, with the presence of branched processes that continuously monitor the surrounding parenchyma [8]. Following the recognition of infection or injury, microglia rapidly shift to an activated proinflammatory state (M1 phenotype) that is tightly regulated in order to limit neuronal damage; after the induction of the immune response, microglia shift to the alternative activated phenotype (M2 phenotype) [9,10] that promotes wound healing and functional recovery. In the context of neuroprotection, the M2 phenotype prevents the degradation and promotes the deposition of extracellular matrix [11] as well as the expression of antiinflammatory cytokines and growth factors [12]. However, the M2 phenotype may also be detrimental for the brain. For example, in glioma-infiltrating microglia, the immune function appears to be suppressed by the acquisition of the M2 phenotype [13], which has protumoral effects.

Hallmarks of normal brain aging [14], often occurring in the absence of neurological diseases, are cognitive impairments, especially deficits in memory, and learning. With aging, brain neurons decrease in number. In some areas, the cell loss is limited, but in other areas such as the hippocampus it amounts to 10–60% [15]. In the cortex, the amount of neuronal cell loss is variable [16,17]. Likewise, microglia are subject to senescence deterioration, with abnormal morphological features. Microglia affected by structural changes typical of senescence are designated as dystrophic microglia, whose morphological characteristics differ from those of activated or quiescent microglia. The most common form of dystrophic microglia is a deramified morphology characterized by the absence of branched cytoplasmic processes and, occasionally, by the formation of spheroids. Other cells may show abnormally tortuous cytoplasmic processes that are shorter than those of the ramified cells. However, the most striking sign of microglial dystrophy is the partial or complete fragmentation of the cell’s cytoplasm [18].

Among the multiple and complex events that occur during brain aging, the role of the TREM2-DAP12 axis, a signaling complex expressed exclusively in microglia, may be fundamental, given that, by regulating the phagocytic function of microglia and the overall fitness of microglia during their lifespan, TREM2 signaling is essential for keeping CNS tissue homeostasis [19]. However, although it is involved in many typical pathologies of the aging brain, little is known about TREM2 physiological role in aging.

CX3CL1-CX3CR1 is a critical signaling pathway for microglia-neuron cross-talk [20,21], yet its role during aging, or how it changes during life, is not fully understood. In the adult brain, CX3CL1-CX3CR1 signaling regulates cognitive functions and synaptic plasticity, particularly in the hippocampus, and neural CX3CL1 expression decreases in the aged rodent brain relative to the young brain. Although the development of neurodegeneration and normal aging are two closely linked processes, the molecular basis of the switch that triggers the transition from healthy aging to neurodegeneration remains unrevealed.

## 2. Microglia in the Aged CNS

The brain shows an increasing inflammatory state with aging. This process is evidenced by the presence of oxidative stress, the increased production and release of proinflammatory mediators, and the development and progression of neurodegenerative diseases [22,23]. This altered inflammatory brain profile is linked to alterations of microglia function [24]. Indeed, there is evidence that microglia in the human brain undergo cellular senescence during aging, which limits microglia ability to proliferate in response to challenges [25], dramatically affecting the capacity of the brain to elicit specific microglial responses, with a bias toward the M1 phenotype and away from the M2 phenotype [26]. Thus, the progressive brain inflammatory state and microglial senescence that occur in the aging brain are likely to be two linked events.

Extensively distributed senescent and age-related dystrophic microglial cells have been shown throughout the brain in human subjects [27]. In animal models, morphological features of dystrophic microglia include very specific morphological changes [28,29] unrelated to the classical and alternative phenotypes. It is known that microglia undergo a burst of mitotic activity during injury-induced activation. In rats, every mitotic wave is followed by a wave of programmed cell death as a mechanism to reduce cell numbers back to the steady state level [30]. In general, the activation of microglia imposes a considerable effort on their metabolic machinery such that they can tolerate high levels of activity for a limited period, and activated microglia are prone to die sooner than non-activated cells. Human microglia are exposed to decades of metabolic stress brought about by a multitude of factors, with continuous cycles of mitosis that promote replicative senescence. In vitro studies have shown that rat and human microglia undergo senescence by progressive telomere shortening [31,32]. Therefore, the deterioration of microglia could be crucial for the development of aging-related neurodegenerative disease.

Microglia are efficient sensors of changes in the CNS microenvironment, and their neuroprotective role has been hypothesized to be impaired during aging [33]. For instance, there is an increased expression of the interleukin (IL)-6 receptor [34], which has been associated with deficits in cognitive function, and aged animals differ from young animals in physiology, pathophysiological features, and behavioral outcome after brain injury [35]. Aged brain microglia are polarized to the M1 phenotype, with a long-lasting impairment of M2 responses after ischemic injury [36,37]. The capacity of microglia to contribute to myelination, promote differentiation of oligodendrocytes, and subsequent remyelination, is significantly reduced in the aged brain [38]. There are also major alterations in ion channels, essential regulators of microglia functions [39]. The hippocampus is selectively vulnerable to age-related neuroinflammation [40]. All these observations support the hypothesis that increased inflammatory responses during aging are at least partially the result of alterations in microglia activation and function [41]. However, microglia degeneration in the human brain is a progressive process that takes place slowly over time and is characterized by a varying degree of structural (i.e., morphological) alterations. Ultimately, severe alterations render microglia fully nonfunctional, with important consequences for brain homeostasis, i.e., phagocytosis impairment and cytokine release.

Emerging evidence indicates that inflammation during aging can modify neuron-astrocyte-microglia interactions, and this mechanism may be involved in neurodegenerative diseases [42]. Microglia actively participate in the death of dopaminergic neurons in Parkinson’s disease (PD), forebrain neurons in Alzheimer’s disease (AD) and motor neurons in ALS [43,44], and an age-related increase in microglia number in the human sub-ependymal zone coincides with decreased cell proliferation and neuronal differentiation [45]. After all, PD, AD, and ALS are typical disorders of aging, and microglia play a major role in these neurodegenerative diseases.

## 3. TREM2-DAP12 Signaling

TREM2 is a member of the Triggering Receptor Expressed on Myeloid Cells transmembrane glycoproteins, which belongs to the single immunoglobulin variable (IgV) domain receptor family [46]. The genes encoding *TREMs* localize to human chromosome 6p21 [47]. The *TREM* cluster includes genes encoding *TREM1*, *TREM2*, *TREM4*, *TREM5* as well as *TREM*-like genes [47]. TREM2 is a glycoprotein of about 40 kDa, which is reduced to 26 kDa after *N*-deglycosylation. The TREM2 gene encodes a 230 amino acids (aa) protein consisting of an extracellular domain, a transmembrane region, and a short cytoplasmic tail. The extracellular region, encoded by exon 2, is composed of a single type V Ig-SF domain, containing three potential *N*-glycosylation sites. The putative transmembrane region contains a charged lysine residue [46,48]. The cytoplasmic tail of TREM2 lacks signaling motifs, therefore a separate signal transduction subunit is required to mediate TREM2 activating signals [48]. The two best characterized members of this receptor family, TREM1 and TREM2, act through association with a DAP12-mediated pathway for signaling [48,49].

DAP12 is a member of the type I transmembrane adapter protein family [50]. Proteins of this family share many structural and functional characteristics, including one or more Immunoreceptor Tyrosine-based Activation Motifs (ITAM) in their cytoplasmic domain, charged acidic residues in the transmembrane region (critical for the interaction with their partner chain), and the ability to recruit src homology domain-2 (SH2)-containing proteins following tyrosine phosphorylation. Human *DAP12* gene maps on chromosome 19q13.1, and DAP12 is a disulfide-bonded, homodimeric polypeptide of 113 aa composed of a 27-aa leader, a 14-aa extracellular domain, a 24-aa transmembrane segments, and a 48-aa cytoplasmic region [51]. The short extracellular domain contains two cysteine residues (Cys33 and Cys35) which allow homodimer formation [52]. The association of DAP12 with its receptors is coordinated by the charged aspartic acid residues located near the center of the transmembrane domain in the DAP12 dimer [53]. Within the cytoplasmic region, DAP12 has a classical ITAM motif that represents the only signaling domain of this polypeptide and mediates all the known effector functions of DAP12 [50,51]. The ITAM is rapidly phosphorylated on two tyrosine residues by Src protein tyrosine kinases, thus providing a docking site for the SH2 domains of other kinases, such as spleen tyrosine kinase (Syk) [54]. Although TREM2 bind ligands, they are unable to trigger intracellular signaling on their own. Signal transduction is carried out by the ITAMs of DAP12, which are absolutely critical for TREM2 signaling [55].

TREM2 mainly controls the function of three cell types, all derived from the myeloid lineage: microglia, osteoclasts, and immature dendritic cells (DCs). The functional effects of TREM2 stimulation were first described in DCs [56]. In microglial cell lines, previous studies [57] have shown that TREM2 expression is essentially intracellular, localizing to the Golgi complex. After ionomycin stimulation, rapid TREM2 cell surface expression was observed, suggesting that the intracellular pool of TREM2 can rapidly translocate to the cell surface.

**Figure 1:**
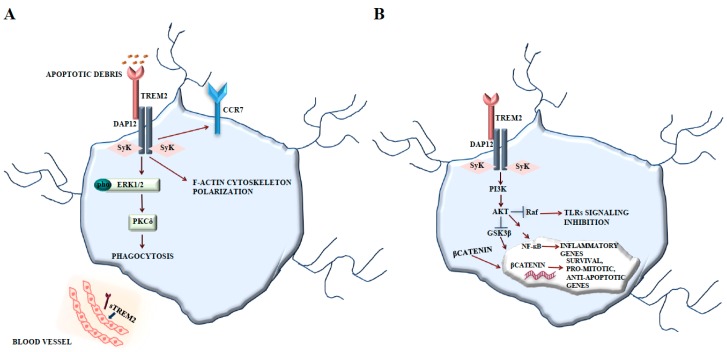
TREM2-DAP12 signaling. (**A**) TREM2 signals via DAP12 and stimulates the protein tyrosine kinase ERK, regulating actin polymerization and cytoskeleton organization. TREM2 upregulates the cell surface expression of the chemokine receptor CCR7 and promotes chemokine-directed migration towards CCR7 ligands. TREM2 activation also stimulates microglial phagocytosis via ERK. (**B**) TREM2 promotes microglial survival by activating Wnt/β-catenin signaling. TREM2 interacts with DAP12 and activates PI3K/Akt signaling, thereby inactivating GSK3β and stabilizing β-catenin that translocates into the nucleus, thereby activating pro-mitotic and anti-apoptotic genes. PI3K/Akt pathway activation by TREM2-DAP12 contributes to the regulation of NF-κB and to the inhibition of TLR signaling by blocking MAPK signaling at the RAF level.

In microglia, TREM2 signals via DAP12 and stimulates the protein tyrosine kinase ERK (Figure 1A) [58]. The activation of TREM2 by antibodies triggers changes in actin polymerization and cytoskeleton organization. Furthermore, the stimulation of TREM2 upregulates the cell surface expression of the chemokine receptor CCR7 and promotes chemokine-directed migration towards the chemokine receptor ligands, i.e., the chemokines CCL19 and CCL21 [58]. TREM2 activation by antibodies also stimulates microglial phagocytosis via ERK. Following the knockdown of microglial TREM2, cells showed reduced capacity to phagocytose apoptotic neural cell membranes [58], and, in this case, TREM2 was also involved in antiinflammatory signaling. Challenging *Trem2*^‒/‒^ microglia with apoptotic neural cells induces increased gene transcription of proinflammatory cytokines compared to microglia having intact TREM2 [58]. Thus, TREM2 appears to counterbalance the proinflammatory activity of microglia. The antiinflammatory signaling of TREM2 in microglia would be in line with a more recently described inhibitory signaling function of the ITAM-containing adaptor molecule DAP12 [59].

A recent work demonstrated that TREM2 promotes microglial survival by activating the Wnt/β-catenin signaling pathway [60], which plays essential roles in many biological processes [61]. TREM2 activation leads to DAP12 phosphorylation via Src family kinases, initiating downstream signaling cascades including PI3K, PKC, and ERK [62]. Conversely, *Trem2* deficiency leads to a dramatic downregulation of Wnt/β-catenin signaling, but not of other kinases, together with decreased microglial survival and enhanced cell death [60]. TREM2 interacts with DAP12 and activates PI3K/Akt signaling, thereby inactivating GSK3β and stabilizing β-catenin (Figure 1B). This cross-talk is a common tract in CNS and has been also described in the astrocyte lineage [63]. GSK3β inhibition determines the accumulation and stabilization of β-catenin that translocates into the nucleus, where it engages transcription factors, driving the expression of survival, pro-mitotic, and anti-apoptotic genes [64]. Understanding whether TREM2 mediates microglial function through the modulation of the Wnt/β-catenin pathway is of great interest. Moreover, PI3K/Akt pathway activation by TREM2-DAP12 contributes to the regulation of nuclear factor-κB (NF-κB) and to the expression of inflammatory genes. The PI3K/Akt pathway can also inhibit toll-like receptor (TLR) signaling by blocking MAPK signaling at the RAF level [50,65].

### 3.1. Physiological Role of the TREM2-DAP12 Axis

The full understanding of the biology of the TREM2-DAP12 complex depends on the identification of TREM2 ligands, however, while it is well known that any role played by TREM2 is carried out by the ITAMs of DAP12, a first unresolved issue relates to the physiological, yet unknown, ligands of TREM2. Many endogenous and exogenous potential ligands for TREM2 have been reported [49,65], such as lipopolisaccaride (LPS), Gram-positive and Gram-negative bacteria, as well as unidentified ligands. Moreover, TREM2-DAP12 plays an important role not only in immune responses, but also in other biological processes, such as brain homeostasis and osteoclastogenesis [66].

Regarding the role in the immune response, human TREM2 regulates the activity of microglia, macrophages, and DCs, playing a critical role in inflammatory response finalization, i.e., inflammatory or antiinflammatory. In this regard, TREM2-DAP12 signaling regulates the cell surface expression of T cell stimulatory molecules and promotes partial DC survival and maturation (inflammatory role) [56]. On the contrary, studies by Ito and Hamerman [67] showed that the TREM2-DAP12 complex plays a negative role in TLR responses of bone marrow-derived DCs (antiinflammatory role).

TREM2 is a key negative regulator of autoimmunity and plays a role in the inhibition of IL-6 and tumor necrosis factor (TNF) production by macrophages and, as mentioned previously, is responsible for DAP12-induced inhibition of inflammatory responses driven by TLR agonists in mouse and human macrophages. Helming et al. [68], by using human and mouse macrophages from *Dap12* defective-mice, showed decreased macrophage fusion and formation of multinucleated giant cells. The same results were obtained by the knockdown of *Trem2* in mouse bone marrow-derived macrophages with severely decreased fusion. They concluded that signaling through the TREM2-DAP12 complex triggers the macrophage differentiation pathway, resulting in the programming of macrophages into a fusogenic state. Contrariwise, studies by Turnbull et al. [69] and Hamerman et al. [70] showed that inflammatory cytokine production increased in *Dap12^−/−^* and *Trem2^−/−^* macrophages activated by TLR agonists. However, the explanation of how TREM2 can provide both inhibitory and activating signals in macrophages remains an open issue.

TREM2-DAP12 also plays a positive role in phagocytosis, binding both endogenous and exogenous ligands. TREM2 expression on both microglia and macrophages is associated with a specific activated cell phenotype, which performs important protective functions, such as the promotion of tissue repair, the control of local inflammation, or the phagocytosis of dying cells [71]. In mouse microglial cells, the knockdown of TREM2-DAP12 reduces the phagocytosis of apoptotic neurons and causes an increase in TNF-α and NO synthase-2 (NOS2) transcription, whereas the overexpression of TREM2 increases phagocytosis, induces cytoskeletal reorganization, reduces TNF-α and NOS2 production, and intensifies the phagocytosis of neuronal debris [58]. TREM2 can also recognize and bind to several species of fungi and bacteria and, in association with DAP12, can promote their phagocytosis [72,73]. These results suggest that TREM2 promotes the innate immune function by binding to exogenous pathogens or to endogenous, not pathogenic ligands. However, the TREM2-DAP12 complex seems to be more engaged in the inhibition than in the activation of the innate immune system, even if the complex’s core activities may lie beyond immune system regulation.

In the CNS, TREM2 and DAP12 form a signaling complex expressed exclusively on microglia and sustain many transcriptional programs that do not suit the conventional M1 and M2 phenotypes [57]. How TREM2 supports these programs remains unclear. However, TREM2 signaling has a variable impact in different brain regions, probably depending on their myelin content, their levels of TREM2 expression, and the presence of alternative TREM2 ligands. In the cuprizone model, which mainly affects the corpus callosum, TREM2 was required for microglial response to prolonged demyelination, for the removal of damaged myelin sheaths, and for the secretion of trophic factors that support oligodendrocyte precursors’ differentiation [38]. Previous work demonstrated that TREM2 is a lipid sensor that may be activated by different stimuli promoting microglia’s health and expansion during normal aging [74]. TREM2 may bind a broad array of phospholipids and glycolipids, which may be exposed on damaged myelin sheaths as well as on dying glial cells and neurons, even if it is unclear whether TREM2 binds lipid components within lipoproteins, exosomes, apoptotic cells, or other supramolecular structures.

TREM2 expression in microglia promotes phagocytosis of apoptotic neurons, producing very small quantities of proinflammatory cytokines. TREM2 is highly expressed in nonstimulated microglia, and its expression strictly overlaps with DAP12 gene expression [75] and is downregulated by LPS and interferon (IFN)-γ [76]. Only microglial clusters and neuron-associated microglia [77] express TREM2. Apoptotic neurons induce undue proinflammatory responses and microglial activation leading to neuroinflammation, if TREM2 is not expressed. Moreover, in the CNS, DAP12-deficient mice have reduced myelination and synaptic degeneration [78]. The exact molecular mechanism of neural degeneration in *Dap12^−/−^*-deficient mice is not fully understood. In vitro data allow speculating that the TREM2-DAP12 complex is fundamental in microglia for the removal of apoptotic cells and other organic macromolecules. A defect of the TREM2-DAP12 complex would lead to the accumulation of toxic products that might directly cause brain damage or overstimulate the microglial cells to release cytotoxic mediators. However, it is unclear which nervous tissue-derived ligand binds to the TREM2.

Another poorly understood aspect of the biology of the TREM family is the release of soluble variants of the receptors, sTREM, whose origin is controversial. Soluble forms have been described for TREM1, TREM2, and TREM-like transcript-1 only. sTREMs were detected in the biological fluids of patients and animals suffering from a variety of diseases and infections, and their presence often correlated with disease severity [79]. Recent studies demonstrated that sTREM2 is released from the surface of cells through the consecutive action of disintegrin and metalloproteinase domain-containing protein 10 (ADAM10) and γ-secretase, or it might originate by alternative splicing of TREM2, leading to secreted TREM2 [80,81]. Thus, sTREM2 might also contribute to the transport, delivery, or metabolism of myelin products.

### 3.2. Pathological Role of the TREM2-DAP12 Axis

**Figure 2:**
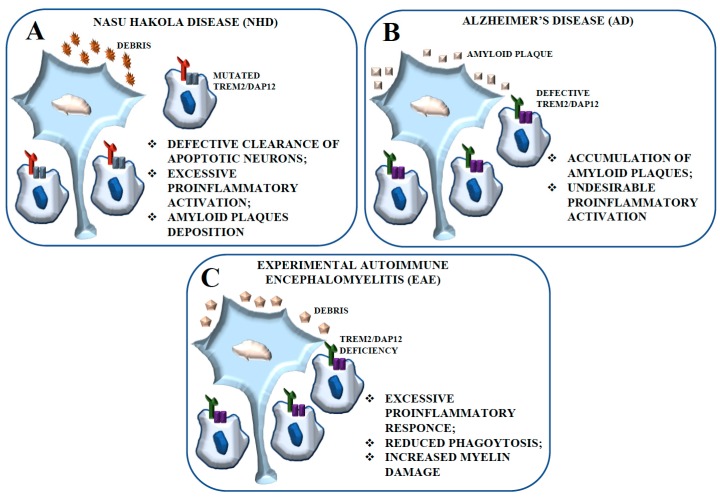
Schematic representation of the contribution of the TREM2-DAP12 pathway to NHD, AD, and Experimental Autoimmune Encephalomyelitis (EAE). (**A**) The defective expression of TREM2 and DAP12 in microglial cells is considered tightly related to the inhibition of apoptotic neuron clearance observed in NHD. Impaired phagocytosis could be responsible for the excessive proinflammatory activation of microglial cells resulting in neurodegeneration. (**B**) The lack of function of TREM2, -DAP12, or both in AD patients leads to failing amyloid engulfment, determining an amyloid-associated dementia. These conditions would cause accumulation of amyloid-β plaques with secondary and undesirable proinflammatory microglial activation and proinflammatory activation. (**C**) The EAE model is an autoimmune demyelinating disorder characterized by the destruction of myelin proteins that recapitulates several aspects of the human disease multiple sclerosis (MS). The lack of function of TREM2, DAP12, or both results in an excessive inflammatory response, with decreased phagocytosis by microglial cells and increased myelin damage.

The term microgliopathy refers to several pathologies whose main feature lies in functional alterations of microglia. Nasu-Hakola disease (NHD) is considered a primary microgliopathy with sclerosing leukoencephalopathy. NHD is a rare intractable autosomal recessive leukodystrophy [82] caused by genetic mutations of either *TREM2* or *DAP12* genes [75]. In the late phase of disease, patients progress to dementia and generally die in the fifth decade of life. Brain magnetic resonance images and computed tomography suggest an activated phenotype in the frontal and temporal white matter in NHD patients [83]. Defects in DAP12 or TREM2 function in NHD microglia result in defective clearance of apoptotic neurons and are believed to play a central role in NHD pathogenesis [81,84] (Figure 2A). TREM2 or DAP12 deficiency may lead to excessive proinflammatory microglial activation which causes neurodegeneration with amyloid plaque deposition. Given the strong evidence supporting the role of TREM2 and DAP12 as inhibitors of TLR signaling [67], it is possible to hypothesize that amyloid-stimulated TLR activation is disinhibited in NHD, with consequent excessive microglial activation and reduced ability to phagocytose amyloid deposits or apoptotic neurons.

TREM2 is strongly expressed in AD brain tissue, notably close to amyloid plaques, and can mediate amyloid plaque phagocytosis. The lack of function of TREM2, DAP12, or both in AD patients leads to failing amyloid engulfment, determining a similar amyloid-associated AD-type dementia as that observed in NHD [85]. These conditions would cause the accumulation of amyloid-β plaques, with secondary and undesirable proinflammatory microglial activation. A similar process occurs in the cuprizone model in which defects in TREM2 function result in the accumulation of myelin debris [86] that continuously recruit nonengulfing microglia (Figure 2B).

Recent studies demonstrated that the heterozygosis for a rare R47H mutation of *TREM2* confers high risk for developing AD [87,88], PD, and sporadic ALS [89,90]. This variant affects TREM2 lipid recognition [91]. Accordingly, it is possible to speculate that defective TREM2-dependent mechanisms, mostly defective phagocytosis, might be a common feature of demyelination, AD, PD, and ALS.

The EAE model is an autoimmune demyelinating disorder characterized by the destruction of myelin proteins that recapitulates several aspects of the human disease MS (Figure 2C). Piccio et al. [71] described a rapid TREM2 upregulation in the CNS at the peak of the disease and its partial downregulation later on, which suggests a role for TREM2 during the early and chronic inflammatory phases of EAE that involve regulatory mechanisms of control of excessive inflammation. However, TREM2 microglial subpopulations that emerge during EAE could have regulatory roles and be immunosuppressive or even tolerogenic, thereby controlling autoimmune responses and the severity of CNS inflammation [92]. In this same study [71], the blockade of TREM2 activation in vivo resulted in a much more severe disease. The worse clinical course of EAE could result from an excessive inflammatory response, with decreased phagocytosis by microglial cells and increased myelin damage. Other studies showed that upon TLR-dependent activation, cytokine production was significantly increased in TREM2-DAP12-deficient mice, indicating that TREM2 either inhibits or counteracts TLR-induced inflammatory responses [69,74]. More generally, an efficient apoptotic cell phagocytosis limits direct tissue injury by preventing the spillage of the potentially harmful contents of the dying cells and by inhibiting secondary immune responses to moieties leaking from apoptotic surface structures. This tissue response is typically associated with the production of antiinflammatory cytokines as well as growth factors involved in tissue repair. TREM2 expression on microglial cells and macrophages in the CNS correlates with a specific activated cell phenotype that exerts important protective functions, such as the phagocytosis of dying cell, the control of local inflammation, and the promotion of tissue repair.

sTREM2 was found to be significantly elevated in the cerebrospinal fluid (CSF), but not in plasma [93], of MS, AD, and frontotemporal dementia (FTD) subjects compared to subjects with noninflammatory neurologic diseases [94].

The release of sTREM2 could represent a mechanism to counter-regulate their activity and could derive from TREM2^+^ cells within the CNS. Accordingly, sTREM2 may act as a soluble “decoy” receptor for the endogenous ligand, effectively inhibiting the engagement of the ligand for the TREM2. This hypothesis seems to be the most cogent and may suggest an additional post-translational regulation of TREM2 activity. Moreover, this post-translational regulation might help understand why TREM2 protein is not readily detected in adult microglia despite the abundant expression of TREM2 mRNA [81,95].

Although sTREM2 is not likely to be a diagnostic marker for MS, it might have a role in disease pathogenesis. If this is true, therapeutic opportunities to increase cell-surface TREM2 expression may be considered. Studies of subjects with dominantly inherited AD have shown that CSF TREM2 elevation can occur approximately 5 years before the onset of symptoms [96]. The effects of *TREM2* genetic variants on CSF sTREM2 do not show a consistent trend. Yet, in MS patients, treatment with the immune modulator natalizumab was shown to decrease sTREM2 to normal levels, suggesting that sTREM2 may have a potential utility as a biomarker [97] that indicates not yet pathological conditions or disease remissions.

In addition to R47H mutation of TREM2, several other deletions or homozygous mutations in *TREM2* have been detected in different families showing a behavioral variant form of FTD [98,99]. The heterozygous expression of *TREM2* variants has also been linked to cases of PD, ALS, and FTD other than AD [89,90]. However, other studies have not associated TREM2 with these neurodegenerative diseases [100,101]; after all, the heterozygosis of *TREM2* variants might contribute to the risk of neurodegenerative diseases other than AD.

### 3.3. The TREM2-DAP12 Axis in Aged Microglia

**Figure 3:**
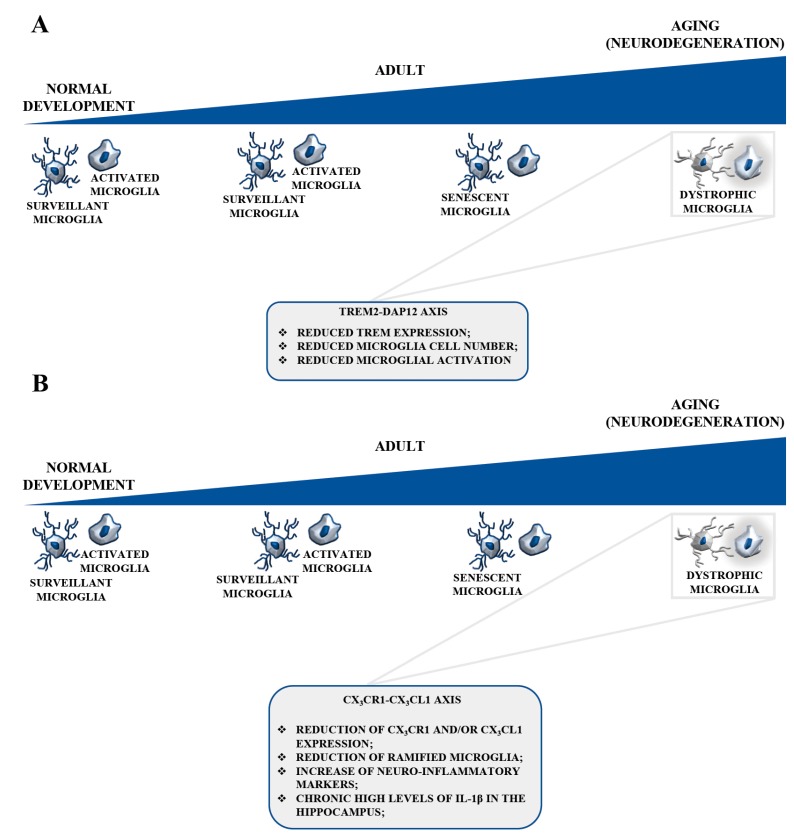
TREM2-DAP12 and CX3CL1-CX3CR1 signaling in aged microglia. (**A**) TREM2 expression is reduced in aged mice. A progressive decline in TREM2 expression might occur during aging in certain areas of the human brain, reducing microglial cell numbers and the microglial response to the breakdown of astrocytes, oligodendrocytes, neurons, and myelin. These events would create a fertile ground for neurodegeneration. TREM2 deficiency hinders the accumulation of microglia during aging. (**B**) CX3CL1 expression levels physiologically decrease in aged rodents which show a reduction of ramified microglia and a rise of neuroinflammatory markers. Reduced levels of CX3CL1 and CX3CR1 in the aged brain greatly alter the overall functionality of this pathway, with morphological and functionally impaired microglia. During aging, reduced neurogenesis occurs in the hippocampus. The pharmacological knockout or genetic deletion of CX3CR1 lead to decreased neurogenesis in the dentate gyrus of the mouse hippocampus, with an IL-1β-dependent decline of both the survival and the proliferation rate of the neuronal progenitors. The physiological decline in neuronal CX3CL1 might contribute to increased microglia activation.

The function of TREM2-DAP12 during aging and in aged microglia remains to be fully understood. It is well known that TREM2 is required to sustain microglial trophic function in the aging brain [38] and that microglial TREM2 expression is reduced in aged mice [102]. Moreover, age-matched *Trem2^−/−^* mice had fewer microglia in areas of demyelination [103] (Figure 3A). It is possible that a progressive decline in TREM2 expression might occur during aging in certain areas of the human brain, reducing microglial cell numbers and the microglial response to the breakdown of astrocytes, oligodendrocytes, neurons, and myelin. These events would create a fertile ground for neurodegeneration. On the contrary, a previous study on sensory systems demonstrated that the total number and density of microglia may increase with age in the sensory cortex, and this would be accompanied by changes in both distribution and morphology and by sensory deficits [104]. Indeed, TREM2 deficiency hinders the accumulation of microglia during aging, particularly in the corpus callosum that is rich in myelin, which might provide a major stimulus for TREM2 signaling. Thus, quantitative analyses of TREM2 expression in different areas of the mouse and human brain at different ages are essential to understand if the decreased TREM2 expression in certain areas is correlated to a greater susceptibility to neurodegeneration during aging or disease.

TREM2-mediated signaling plays a role in microglial proliferation and survival in response to aging, although controversial results have been obtained. No significant discrepancies in the microglia number between *Trem2^−/−^* and wild-type (WT) mice until two years of age were detected [38], while, during demyelination in *Trem2^−/−^* mice [103], microglia proliferation decreased, indicating a key role of TREM2 in microglia proliferation and survival. Other studies demonstrated that a lack of TREM2 in osteoclast precursors prevented proliferation and β-catenin activation [105], and DAP12 signaling deficiencies in aged mice resulted in a remarkable reduction of microglia number [106]. Regarding the reduced expression of TREM2 during aging described by Hickman [102], it is interesting to note that this reduction was accompanied by an overall increase in the expression of genes involved in neuroprotection, with downregulation of the neurotoxic phenotype. Yet, several studies have presented conflicting results [107].

Apolipoprotein E (APOE) and TREM2 have been recently involved in the induction of a unique signature in microglia [107]. In a homeostatic environment, the gene expression pattern of microglia is mainly controlled by TGF-β signaling with low APOE expression, whereas in aging and in pathological conditions such as AD, MS, and ALS, the microglia phenotype is characterized by high APOE expression with activation of TREM2 signaling consequent to the phospholipid-APOE complex exposure [107] on apoptotic neurons [91,108]. This microglia signature is maintained across aging and diseases, but following TREM2 or APOE knockout, the microglia response to brain tissue damage is seriously blunted. This work identifies the TREM2-APOE axis as a master regulator of the microglial functional phenotype in aging and neurodegenerative diseases, inducing a microglia phenotypic switch from a homeostatic to a neurodegenerative phenotype. The modulation of the microglial neurodegenerative phenotype through targeting of the TREM2-APOE pathway might serve as a way to restore a homeostatic microglia and treat neurodegenerative disorders.

Microglia exhibit various priming states that determine their responses to subsequent injury or infection of the brain [109,110]. Such priming states are characterized by increased expression of a defined set of genes that regulate microglial behavior [109,110]. Aged microglia is a priming state that shows a characteristic expression pattern. In microglia isolated from young (5 months) versus old (24 months) mice, there is a shift of the microglial phenotype towards an alternative neuroprotective priming state [102]. In this context, the complete expression pattern of microglia, not clearly correlated to the classical or alternative phenotype, showed a significant downregulation of genes encoding proteins involved in sensing apoptotic neuron, among which *Trem2*, whereas genes encoding proteins involved in sensing endogenous ligands were significantly upregulated [102]. Confirming that aged microglia exhibit a unique gene expression signature, Krasemann et al. [107] performed a gene-set enrichment analysis. The results showed that an overlap exists between aged microglia phenotype, phagocytic microglia, and microglial molecular signatures typical of models of ALS, AD, and lipid disorders. This typical signature of aged microglia includes the TREM2-DAP12 and probably the CX3CL1-CX3CR1 pathways. The prevalence of any of these molecular signatures could give rise to neurodegenerative pathologies or physiological aging. From this perspective, the aged microglia would prime towards a variable destiny with the predominance of physiological aging or pathological disorders, as a result of genetic or epigenetic risk factors. Aging itself is the most significant risk factor for AD and other neurodegenerative diseases and is associated with profound changes in the brain parenchyma, such as enhanced microglia activity and reactive gliosis. 

In healthy controls, a positive correlation between CSF sTREM2 levels and age was detected with almost a three-fold increase from 50 to 90 years of age [111]. These data suggest that sTREM2 levels are linked to the normal, nonpathological aging process. Similarly, *TREM2* mRNA increased by 50–100% from 50 to 90 years of age with no evident pathological processes [112]. Similar to the aging brain, TREM2 protein and mRNA levels are elevated in AD brain [113]. On the contrary, sTREM2 levels are elevated in aging but not in AD brain [111]. Therefore, different mechanism might concur to the regulation of sTREM2 expression, depending on aging and diseases. Another interesting relationship is between CSF sTREM2 and CSF Tau protein levels, with a three-fold increase in Tau protein from 40 to 90 years of age in physiological aging [114,115]. Indeed, asymptomatic tauopathy has been described during aging [116]. A similar relationship was described between CSF sTREM2 and CSF Amyloyd-β peptide during aging in the absence of amyloid deposit, likely reflecting a very early stage of dementia [117,118].

## 4. CX3CL1-CX3CR1 Signaling

CX3CL1 is the only member of the δ subfamily of chemokines and it unusually appears to bind only one receptor, the seven transmembrane G_i_ protein-coupled CX3CR1 [119]. Many other chemokine members exhibit more promiscuous binding activity than CX3CL1 [119]. The full-length molecule is larger than most other chemokines [120] and exists in two distinct forms. The 95 kDa full-length membrane-bound form possesses a 76-aa N-terminal chemokine domain, a 241-aa glycosylated mucin-like stalk, an 18-aa hydrophobic transmembrane region, and a 37-aa intracellular C-terminal domain [120,121]. The second form is an approximately 70 kDa soluble form that contains the N-terminal chemokine domain and the extracellular mucin-like stalk [120,121]. The soluble chemokine domain of CX3CL1 acts as a signaling molecule and may bind microglial-expressed CX3CR1 receptors [122], whereas its membrane-tethered mucin stalk can serve as a cell adhesion molecule for microglia and infiltrating leucocytes during an inflammatory episode [123,124]. In the CNS, CX3CL1 is constitutively and abundantly expressed in neurons [125], with particularly high levels in hippocampal neurons [125], while in astrocytes it can be induced by TNF-α and IFN-γ treatment [126]. 

CX3CR1 activation is linked to several intracellular second messengers. In the brain, CX3CR1 expression is restricted to microglia [125]. In microglia, activation of the CX3CL1-CX3CR1 axis by both soluble and membrane-bound CX3CL1 was shown to decrease LPS-induced MHCII, CD40 mRNA levels, and IL-1β protein expression, and these antiinflammatory effects were shown to be Akt- and PI3-K-dependent [127]. CX3CL1-CX3CR1 also rapidly increases Akt activation in microglia in a dose- and time-dependent manner.

**Figure 4:**
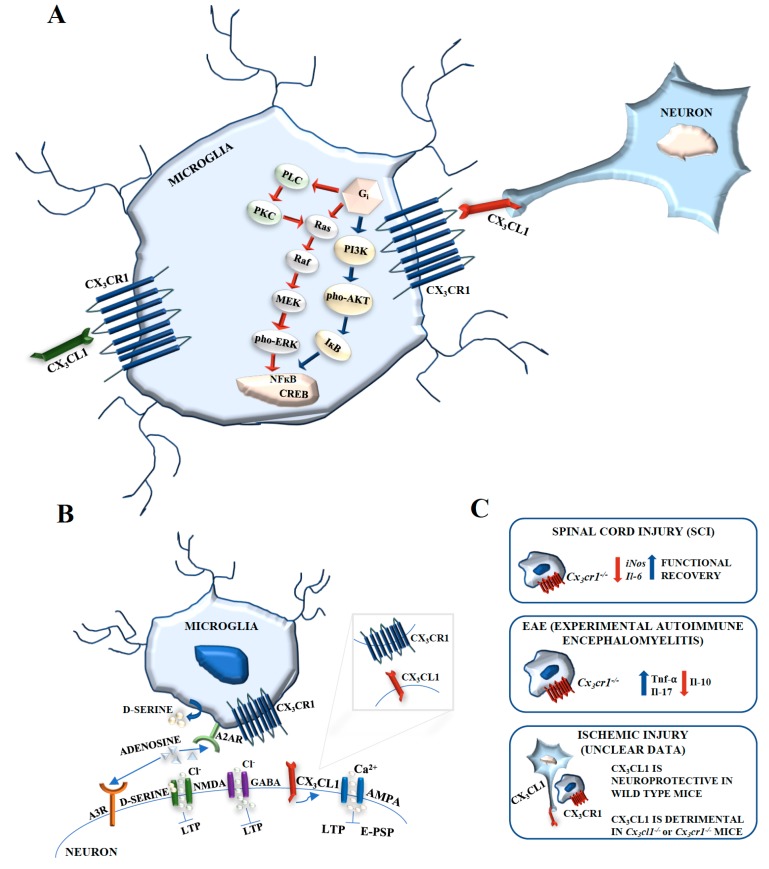
CX3CL1-CX3CR1 signaling. (**A**) CX3CL1 induces transient phosphorylation of Akt and ERK1/2. Soluble CX3CL1 activates the transcription factor CREB and ERK1/2 and induces the translocation of NF-κB p65 subunit to the nucleus. NF-κB p65 subunit nuclear translocation is avoided by a specific inhibitor of PI3-K, suggesting that CX3CL1-CX3CR1 activates NF-κB through Akt. (**B**) CX3CL1, acting through CX3CR1, modulates α-amino-3-hydroxy-5-metyl-4-isoxazolepropionic acid receptor (AMPA) phosphorylation leading to increased calcium entry and inhibition of both excitatory postsynaptic potentials and long-term potentiation. CX3CL1 can increase inhibitory postsynaptic currents, possibly by enhancing neuronal responsiveness to GABA-mediated chloride entry. CX3CL1 may activate CX3CR1 on microglia with consequent adenosine release that, in turn, could activate A3R receptors on neurons. Adenosine activates A2AR on microglial cells and induces the release of d-serine. The adenosine released by microglia has also been involved in neuroprotection by activating A1R receptor subtypes in neurons. (**C**) CX3CL1-CX3CR1 involvement in spinal cord injury, experimental autoimmune encephalomyelitis, and ischemic injury.

The treatment of primary mixed neuronal-glial cell cultures with CX3CL1 induced transient phosphorylation of Akt within 10 min and of ERK1/2 within 1 min of exposure [128] (Figure 4A). Moreover, CX3CL1 significantly inhibited *N*-methyl-d-Aspartate receptor (NMDA)-induced calcium influx in neurons, and this event was abolished by ERK1/2 signal inhibition. CX3CL1 also inhibited NMDA-mediated apoptosis via Akt and ERK1/2 signaling [128]. This effect was probably mediated by microglia cell activation rather than by a direct effect of CX3CL1 on neurons. The treatment of a pure neuronal hippocampal culture with soluble CX3CL1 activated the transcription factor CREB and ERK1/2, but no JNK or p38 MAPK [129], and also induced the translocation of NF-κB p65 subunit to the nucleus. NF-κB p65 subunit nuclear translocation was avoided by a specific inhibitor of PI3-K, suggesting that CX3CL1-CX3CR1 activated NF-κB through Akt [130] (Figure 4A). However, these results were not confirmed in other experimental model and might be determined by the cell culture system, i.e., microglia contamination.

CX3CL1 acting through CX3CR1 modulates α-amino-3-hydroxy-5-metyl-4-isoxazolepropionic acid receptor (AMPA) phosphorylation leading to increased calcium entry and inhibition of both excitatory postsynaptic potentials and long-term potentiation [131] (Figure 4B). CX3CL1 can also increase inhibitory post-synaptic currents, possibly by enhancing neuronal responsiveness to γ-aminobutyric acid (GABA)-mediated chloride entry [132]. CX3CL1 may activate CX3CR1 on microglia with following adenosine release that in turn, could activate Adenosine (A)3 receptors (R) in neurons, inducing a signaling cascade which results in the modulation of GABA_A_ receptors to increase their sensitivity to GABA [133]. Adenosine may also activate A2AR on microglial cells and induce the release of d-serine which acts as a co-agonist of the NMDA receptor, leading to increased calcium entry [134]. The adenosine released by microglia has also been involved in neuroprotection by activating A1R receptor subtypes in neurons [135] (Figure 4B). 

Several studies demonstrated that CX3CL1 suppresses LPS-induced microglia activation by reducing the production of nitric oxide (NO), IL-6, and TNF-α [127,136] and inhibits neuronal cell death due to LPS-activated microglia in vitro, limiting the release of inflammatory factors [136]. These data suggest that the high level of endogenous CX3CL1 expressed in neurons in the adult CNS leads to a tonic activation of CX3CR1 on microglia and acts as a neuronal off signal maintaining microglia in a quiescent state [137,138], thus mediating a neuroprotective role for CX3CL1-CX3CR1 signaling. Contrariwise, in mixed neuron-glia cultures derived from *Cx3cr1^−/−^* mice and in CX3CR1 silenced BV-2 cells, the LPS-induced release of TNF-α, NO, and superoxide is reduced compared to WT cells, [139] suggesting that CX3CL1 is involved in the release of proinflammatory substances by activated microglia.

### 4.1. Physiological Role of the CX3CL1-CX3CR1 Axis

In physiological conditions, CX3CL1-CX3CR1 signaling is involved in different brain functions, both in development and in adulthood. Recently, a key role in synaptic pruning has been attributed to microglia that phagocytes synapses during mouse brain postnatal maturation [140]. *Cx3cr1*^GFP/GFP^ mice, in which microglia are fluorescent and the Cx3cl1 receptor is knocked out (C*x3cr1*^‒/‒^), possess more synapses than WT mice, at least until the third week of life [140]. In hippocampal CA1 region, *Cx3cr1^‒/‒^* mice also show reduced numbers of microglia during postnatal development, suggesting that CX3CL1 signaling may act as a chemotropic agent to attract microglial cells within the brain [140]. Therefore, knocking out of CX3CR1 would render microglia unresponsive to the attractive action of CX3CL1, reducing microglia number in the brain. Consequently, the high synaptic density in *Cx3xr1*^‒/‒^ mice may be explained by a smaller number of microglia.

CX3CL1 also inhibits neuronal cell migration by increasing neuronal binding to the extracellular matrix [141]. On the other hand, CX3CL1 has the opposite effect on microglial cells. Blocking CX3CR1 and in response to CX3CL1, microglial migration decreases [142]. This supports the hypothesis that the CX3CL1-CX3CR1 axis might act as a guide for the microglia, promoting the colonization of the CNS. Moreover, microglial cell infiltration into the barrel centers of the developing somatosensory cortex, that normally occurs around postnatal day 5, is delayed by a few days in *Cx3cr1*^‒/‒ GFP/GFP^ mice, even if no differences are detected at postnatal day 9 [143].

The absence of CX3CR1 also delays the maturation of functional glutamate receptors [143]. Synaptic maturation is influenced by microglia during development, and the CX3CL1-CX3CR1 signaling contributes to this event.

In the adult brain, CX3CL1, produced by neurons, probably holds back microglia in a quiescent state. Microglial cell activation occurs when levels of CX3CL1 decrease, e.g., in the hippocampus of aged rats [127]. This homeostatic suppression of microglial activation may be the major role of the CX3CL1-CX3CR1 axis.

Strong microglial activation in response to intraperitoneal LPS infusion was evidenced in *Cx3cr1*^‒/‒ GFP/GFP^ mice and, interestingly, the transplantation of these LPS-activated microglia in WT mice was quite different compared with the transplantation of microglia taken from *Cx3cr1*^+/−^ mice [129]. *Cx3cr1*^+/‒^ microglial cells migrated quickly from the site of injection and preferentially invaded white matter tracts, whereas *Cx3cr1*^‒/‒^ microglia were localized to the site of injection. Furthermore, neuronal cell loss surrounding the activated GFP^+^ microglia transplanted from *Cx3xr1*^‒/‒^ mice was more sustained than in WT brains that received *Cx3cr1^+/−^* microglial cells, probably because of an increased production of IL-1β from *Cx3cr1^−/−^* microglia [144].

In a neurodegenerative mouse model of PD [144], *Cx3cr1^−/−^* mice showed increased cell death in the pars compacta of substantia nigra compared to *Cx3cr1^+/+^* PD mice, and the same results were obtained in *Cx3cl1^−/−^* mice, suggesting that the CX3CL1-CX3CR1 axis modulates microglia activity and that alterations of this axis result in microglia perturbation, regardless of which one of them is altered.

In another rat model of PD [145], CX3CL1 was neuroprotective and counteracted neuronal cell death in the striatum. Indeed, CX3CL1 infused into the rat striatum resulted in neuroprotection of neurons and in a marked reduction in activated microglia. Likewise, in vitro cocultures of hippocampal neurons and microglia showed neuronal cell death if microglia were pre-exposed to LPS, and this effect was partially abolished by CX3CL1 treatment [146]. The activation of microglial cells with LPS converted the microglia from a resting state into a phagocytic and neurotoxic phenotype.

### 4.2. Pathological Role of the CX3CL1-CX3CR1 Axis

CX3CL1-CX3CR1 signaling plays an important role in neuroinflammatory and autoimmune diseases of the CNS. MS is perhaps the typical autoimmune CNS disease, characterized by inflammation and demyelinating lesions in the spinal cord and brain [147]. EAE is the best disease model closely related to MS [148], with changes in the expression levels of CX3CL1 and CX3CR1 receptor in and around the demyelinating lesions. Indeed, the accumulation of CX3CR1-expressing microglia within brain lesions and sites of inflammation [149] was detected in rats with induced EAE, without changes in neuronal CX3CL1 levels. It is important to note an increase in astrocyte CX3CL1 expression in the proximity of the sites of inflammation, which suggests that reactive or activated astrocytes may attract microglia to the sites of inflammation [149]. Another aspect relates to the upregulated CX3CL1 in EAE brain microglia [121]. In this context, the upregulation of CX3CL1 expression may be a process by which microglia attempt to return to a quiescent state inhibiting their overactivation. Moreover, EAE-affected *Cx3cr1^−/−^* mice displayed more severe EAE symptoms than wild-type animals. EAE-affected *Cx3cr1^−/−^* mice also displayed overexpression of proinflammatory cytokines, i.e., TNF-α and IL-17, compared to EAE wild-type mice (Figure 4C). By contrast, the levels of the antiinflammatory cytokine IL-10 were significantly higher in EAE wild-type mice compared to diseased *Cx3cr1^−/−^* animals [150] (Figure 4C). These results demonstrate the close relationship between CX3CL1and CX3CR1 in autoimmune regulation. More specifically, autoimmune dysfunction in the CNS may be a trigger of microglia activation. However, although there is ample evidence that microglia activation contributes to neuronal damage in MS, there is also evidence of important reparative functions. Microglia themselves could upregulate CX3CL1-CX3CR1 expression and this may be a mechanism by which microglia attempt to autoregulate their overactivation and return neighboring microglia to a quiescent state. The extent of this autoregulation could push microglia toward neuronal destruction or to a more protective phenotype. Confirming this, in MS, a polymorphic variant of CX3CR1, *Cx3cr1^I249/T280^* [151], was shown to affect CX3CL1 binding affinity and receptor expression.

Spinal cord injury (SCI) causes an important trauma to neuronal cells and results in the complete destruction of axons, leading to inflammation and neurodegeneration at the site of injury and around it [152], followed by the recruitment of microglia and monocyte-derived macrophages [153]. Microglia and macrophages promote the formation of a glial scar, thus contrasting the functional recovery of the injured neurons and decreasing the chance of survival of the whole organism [154]. *Cx3cr1^−/−^* mice show a distinct population of macrophages that infiltrate the injured spinal cord compared to wild-type mice, with peculiar functional properties [155]. *Cx3cr1^−/−^* microglia produced less iNOS and IL-6 mRNAs post-SCI (Figure 4C). Moreover, in *Cx3cr1^−/−^* mice, the functional recovery after SCI was faster and greater, suggesting that neuronal-microglial relations are in dynamic equilibrium during neuronal regeneration. Therefore, in SCIs, *Cx3cr1^+/+^* WT microglia may release factors that activate astrocytes and promote glial scar formation, while restricting the functional regeneration of axons (Figure 4C). CX3CR1 pharmacological inhibition during the appropriate temporal window post-SCI may serve as a novel means to inhibit microglial activation and promote neurodegeneration.

Despite the clear neuroprotective role of CX3CL1-CX3CR1 in CNS, in some circumstances CX3CL1 may be harmful. In *Cx3cl1^−/−^* mice, studies performed to investigate the role of CX3CL1 following ischemic injury have suggested that CX3CL1 expression is detrimental to post-ischemic injury recovery [156] (Figure 4C). A similar study in *Cx3cl1^−/−^* and *Cx3cr1^−/−^* mice, showed that both mouse strains showed a smaller infarction volume following ischemia [157], and, following the administration of exogenous CX3CL1 to WT mice, the volume of ischemic infarct was reduced (Figure 4C). CX3CL1 administration to *Cx3cr1^−/−^* mice had no effects. Moreover, in oxygen-glucose deprivation that in vitro resembles in vivo ischemic conditions, CX3CL1 caused a reduction of TNF-α release from *Cx3cr1^−/−^* microglia. These results possibly explain why exogenous CX3CL1 enhances the infarct volume in *Cx3cl1^−/−^* mice, given that a neuroprotective role for TNF-α has been described [158]. In wild-type microglia, CX3CL1 does not affect TNF-α release.

Furthermore, in *Cx3cr1^−/−^* mice the infarct size post-ischemia was reduced compared with WT and heterozygote mice. An increased IL-1β expression in *Cx3cr1^+/−^* mice compared to knockouts was detected in astrocytes. This probably suggests that in stressed conditions such as an ischemic event, microglia lacking the CX3CR1 receptor assume a phenotype that alters the astrocytic function [159].

An interesting work by Pimentel-Coelho et al. [160] regarding sex-specific CX3CL1-CX3CR1 effects on ischemic injury, showed that twelve weeks post-ischemia in WT and *Cx3cr1^−/−^* mice, WT ischemic females recovered better than *Cx3cr1^−/−^* ischemic females [160]. No difference was detected in males. Consequently, for unknown reasons, CX3CR1 signaling may have a greater neuroprotective role in female compared to male mice in ischemic events.

### 4.3. The CX3CL1-CX3CR1 Axis in the Aged Microglia

New insights are emerging concerning the role of the CX3CL1-CX3CR1 axis in the aged brain. A relevant piece of data is that CX3CL1 expression is high in young rodent brain, and these levels physiologically decreased in aged rodents which show, together with a reduced CX3CL1 expression, a reduction of ramified microglia and a rise of neuroinflammatory markers [161] (Figure 3B). Moreover, aged mice subjected to peripheral LPS injection, amplify the microglia response [37,127,161], confirming an antiinflammatory and protective physiological role of CX3CL1. It is interesting to note that an LPS challenge also induced a decrease in the levels of CX3CR1 in aged compared to young brain, with microglia showing CX3CR1 long-term downregulation [162]. Recent studies have confirmed these results and, whereas in adult mice microglia restored the CX3CR1 level 24 h after LPS treatment, this functional recovery did not occur in microglia of aged mice [37]. This lack of recovery in CX3CR1 expression was accompanied by increased levels of IL-1β expression, with an exacerbation of the disease. Taken together, reduced levels of CX3CL1, CX3CR1, or both in the aged brain greatly alter the overall functionality of this pathway, with morphological and functional impaired microglia.

It is known that, during aging, reduced neurogenesis occurs in the hippocampus. Similarly, pharmacological knockout or genetic deletion of CX3CR1 leads to a decreased neurogenesis in the dentate gyrus of the mouse hippocampus [133,161], with an IL-1β-dependent decline of both the survival and proliferation rate of the neuronal progenitors [161]. In this context, the physiological decline in neuronal CX3CL1 [161] might contribute to increase microglia activation. Whether the same occurs in humans remains to be established. Given the decreased hippocampal neurogenesis in cognitive disorders and aging, further studies are needed to establish the involvement of the CX3CL1-CX3CR1 axis in the aforementioned human pathologies. Microglial CX3CR1 regulates PI3K activity to downregulate IL-1β production [163]. Since aging is characterized by chronically high IL-1β levels in the hippocampus [164], and IL-1β directly acts to inhibit the cell cycle in neural progenitors [165], defective CX3CR1 signaling might contribute to low levels of neurogenesis during aging. These effects are reverted by blocking IL-1β function [161].

Recent work has clarified the IL-1β signaling pathway. Sirtuin 1 (SIRT1) belongs to the sirtuin family of nicotinamide adenine dinucleotide (NAD^+^)-dependent deacetylases [166] and acts on various substrates including NF-κB. SIRT1 has been associated to neuroprotection in several experimental models, and a significant reduction of its protein levels has been detected in aging hippocampus [167]. Moreover, caloric restriction, that counteracts many of the age-induced alterations in the hippocampus, upregulates SIRT1 expression. In primary neuronal cultures, NF-κB signaling induced by amyloid-β peptides is downregulated by SIRT1 overexpression, with a strong neuroprotective effect [168]. CX3CR1 inhibits AMP-dependent protein kinase A (PKA), and deletion of CX3CR1 may facilitate the activation of PKA and the subsequent NF-κB activation [169]. In *Cx3cr1^−/−^* microglia, SIRT1 activity increases [170] most likely to counteract excessive NF-κB activation, however, during brain aging, this increment is insufficient to avoid NF-κB-dependent gene expression [171], as evidenced by the high IL-1β levels in the hippocampus.

Previous studies on different animal models of neurodegeneration, showed that the loss of microglia-neuron relationships consequent to CX3CL1-CX3CR1 signal destruction, results in a greater neurotoxic activity of the microglia and an aggravation of disease [144]. However, whether this CX3CL1-CX3CR1 signaling impairment occurs as a consequence or as a cause of increased microglial activation is unclear, even though, as a result of normal aging, both events could take place. Moreover, Bachsetter et al. [161] have detected no changes in hippocampal CX3CL1 mRNA in aged compared to young rats, demonstrating that post-translational mechanisms cause CX3CL1 downregulation. Exogenous CX3CL1 administration recovered the physiological levels of neurogenesis. In addition, a smaller CX3CL1 decrement was detected in middle-aged rats, with no alterations in CX3CL1-CX3CR1 functions, compared to aged rats, suggesting a direct role of aging in CX3CL1 downregulation. In other words, the apparently physiological CX3CL1 decrease that occurs in aging may be compensated at an early stage, but not when aging proceeds.

If genetic alterations are considered, two common single nucleotide polymorphisms (SNPs) in the coding region of *CX3CR1* were detected. Interestingly, these SNPs are linked to age-related diseases, with an increased risk for age-related macular degeneration [172,173] but with a reduced risk for atherosclerosis [174]. Moreover, the level of plasma-soluble CX3CR1 is significantly greater in patients with mild to moderate AD than in patients with severe AD. Considering that in AD patients the disease severity progresses with aging, this is in agreement with the neuroprotective role of CX3CL1-CX3CR1 [175]. It is worth noting that, in aged mice, voluntary physical exercise increased brain CX3CL1 levels and consequently hippocampal neurogenesis [176], with an improvement of hippocampal functions [177,178], suggesting that reduced physical activity during aging might contribute to the decrease of CX3CL1 levels.

## 5. Conclusions

The traditional point of view that microglia are inactive, unless provoked by insults or pathogens, is not correct and, de facto, microglia inactivity is as dangerous as is excessive activity. During lifetime, the CNS has to ensure an appropriate environment so that microglia and all other cell types can perform their tasks. This appears not to occur in the aging brain. Moreover, genetic alterations and epigenetic factors may contribute to altering the CNS environment with an abnormal microglia behavior.

However, there is still much to fully understand about the role of microglia during aging. Probably, different microglial populations act during life, and, indeed, three different microglia populations have been identified in embryonic and postnatal development [179]. Still other microglial population(s) might be present in the aged brain, with potential involvement in the onset of neurodegenerative disorders. The high performance of recent techniques allows the identification and purification of these functionally different microglia populations and the characterization of their expression profiles [180]. Moreover, transgenic animal models are very performing. To date, the *Cx3cr1CreEr* mouse model, with a Cre recombinase fused to the human estrogen receptor inducible by tamoxifen, enables the study of microglia in adults and during aging [181].

During life, a proper and constant communication between the different brain cell types is needed. These relationships are altered or lost in aging, with the onset of severe pathologies. However, when considering the aging as a physiological process of the final part of life, it remains to be understood why some subjects are affected by neurodegenerative disorders while others are not. There is increasing evidence for the importance of epigenetic and genetic factors as causes of “physiological” or “pathological” aging. If this were true, aging could be controlled and guided by acting on these factors.

Microglia degeneration in the human brain is a progressive process that occurs slowly over time and causes the destruction of relationships with other cell types, such as neurons. Among the signaling pathways required for maintaining correct neuron-microglia relationships, the TREM2-DAP12 and CX3CL1-CX3CR1 axes are key factors.

TREM2 signaling is essential for maintaining CNS tissue homeostasis. TREM2-DAP12-mediated phagocytosis of accidental apoptotic materials is a beneficial function of microglia that takes place without frank inflammation. However, excessive neuronal cell death, i.e., during aging, could generate extensive inflammatory processes with deleterious effects on the brain. Different TREM2-positive phenotypes can be assumed by microglia depending on local and temporal conditions, with or without inflammation, generating protective or detrimental effects.

Similarly, the CX3CL1-CX3CR1 pathway might have either destructive or beneficial potential. Experimental data show that the microglial phenotype reverts from beneficial to detrimental during aging and the progression of neurodegeneration, and, in these conditions, it might affect the CX3CL1 effects via CX3CR1 receptor, with a different outcome. With age, the dialog between microglia and neurons via CX3CL1 seems to be disrupted. Moreover, during inflammation, astrocytes upregulate CX3CL1, thus making a communication network with neuron and microglia possible. CX3CL1-CX3CR1 counteracts neuroinflammation as a whole. This seems important in aged hippocampal neurons, where the physiological decrease in CX3CL1 correlates with the cognitive impairment detected in older animals. However, as in the case of TREM2-DAP12 signaling, local and temporal conditions are fundamental for a proper signaling. In light of the many potential roles of TREM2-DAP12 and CX3CL1-CX3CR1 in brain aging and aged-related diseases, novel therapeutic strategies with pharmacological agents able to induce neuroprotective microglial phenotypes could be envisioned. Such strategies have the potential to robustly impact treatments not only for aging disorders, but also for other neurodegenerative and inflammatory CNS conditions.

## Abbreviations

NHD	Nasu-Hakola disease
AD	Alzheimer’s disease
PD	Parkinson’s disease
CNS	Central Nervous System
TREM2	Triggering Receptor Expressed on Myeloid Cells 2
DAP12	DNAX activation protein 12
IL	Interleukin
ALS	amyotrophic lateral sclerosis
ITAM	Immunoreceptor Tyrosine-based Activation Motif
SH2	src homology domain-2
Syk	Spleen tyrosine kinase
EAE	Experimental autoimmune encephalomyelitis
NF-κB	Nuclear factor-κB
TLR	Toll like receptor
LPS	Lipopolisaccaride
DCs	Dendritic cells
TNF	Tumor necrosis factor tumor necrosis factor
NOS2	NO Synthase-2
IFN	Interferon
ADAM10	Metalloproteinase domain-containing protein 10
FTD	Frontotemporal dementia
CSF	Cerebrospinal fluid
NDMA	*N*-methyl-d-aspartate receptor
GABA	γ-aminobutyric acid
NO	Nitric oxide
AR	Adenosine receptor
SCI	Spinal cord injury
MDMs	Monocyte-derived macrophages
SIRT1	Sirtuin1
PKA	AMP-dependent protein kinase A
APOE	Apolipoprotein E.
